# A Multicenter Evaluation of Trends in Antimicrobial Resistance Among *Streptococcus pneumoniae* Isolates From Adults in the United States

**DOI:** 10.1093/ofid/ofac420

**Published:** 2022-09-02

**Authors:** Salini Mohanty, Kelly D Johnson, Kalvin C Yu, Janet A Watts, Vikas Gupta

**Affiliations:** Center for Observational and Real World Evidence (CORE), Merck & Co., Inc., Rahway, New Jersey, USA; Center for Observational and Real World Evidence (CORE), Merck & Co., Inc., Rahway, New Jersey, USA; Becton, Dickinson & Company, Franklin Lakes, New Jersey, USA; Becton, Dickinson & Company, Franklin Lakes, New Jersey, USA; Becton, Dickinson & Company, Franklin Lakes, New Jersey, USA

**Keywords:** antibiotic resistance, invasive pneumococcal disease, invasive pneumococcal vaccines, macrolides, *Streptococcus pneumoniae*

## Abstract

**Background:**

Management of pneumococcal disease is complicated by high rates of antimicrobial resistance (AMR). This study assessed AMR trends for Streptococcus pneumoniae isolates from adults with pneumococcal disease.

**Methods:**

From January 2011 to February 2020, we evaluated 30-day nonduplicate *S. pneumoniae* isolates from 290 US hospitals (BD Insights Research Database) from adults (≥18 years) in inpatient and outpatient settings. Isolates were required to have ≥1 AMR result for invasive (blood, cerebrospinal fluid/neurologic) or noninvasive (respiratory or ear/nose/throat) pneumococcal disease samples. Determination of AMR was based on facility reports of intermediate or resistant. Descriptive statistics and generalized estimated equations were used to assess variations over time.

**Results:**

Over the study period, 34 039 *S. pneumoniae* isolates were analyzed (20 749 [61%] from noninvasive sources and 13 290 [39%] from invasive sources). Almost half (46.6%) of the isolates were resistant to ≥1 drug, and noninvasive isolates had higher rates of AMR than invasive isolates. Total *S. pneumoniae* isolates had high rates of resistance to macrolides (37.7%), penicillin (22.1%), and tetracyclines (16.1%). Multivariate modeling identified a significant increasing trend in resistance to macrolides (+1.8%/year; *P* < .001). Significant decreasing trends were observed for penicillin (−1.6%/year; *P* < .001), extended-spectrum cephalosporins (ESCs; −0.35%/year; *P* < .001), and ≥3 drugs (−0.5%/year; *P* < .001).

**Conclusions:**

Despite decreasing trends for penicillin, ESCs, and resistance to ≥3 drugs, AMR rates are persistently high in *S. pneumoniae* isolates among US adults. Increasing macrolide resistance suggests that efforts to address AMR in *S. pneumoniae* may require antimicrobial stewardship efforts and higher-valent pneumococcal conjugate vaccines.

## Teaser

Persistently high rates of antimicrobial resistance (AMR) were observed in 34,039 *S*. *pneumoniae* isolates in US adults, and macrolide resistance increased over the study period. Higher-valent pneumococcal conjugate vaccines and antimicrobial stewardship efforts may further reduce S. *pneumoniae* AMR.

Despite the availability of pneumococcal vaccines and antibiotics, which have led to substantial declines in pneumococcal disease, *Streptococcus pneumoniae* continues to exert a heavy burden on individuals and health care systems [1]. In the United States, an estimated 2 million pneumococcal infections occur annually, resulting in >6000 deaths and costs of $4 billion [2]. Pneumococcal infections can be either noninvasive, such as otitis media, sinusitis, and community-acquired pneumonia (CAP), or invasive pneumococcal disease (IPD), which occurs when pneumococcal bacteria enter a sterile site such as the bloodstream (bacteremia) or cerebrospinal fluid (meningitis) [1]. In the United States, >90% of IPD cases are in adults; adults age ≥65 years have the highest rates of IPD and the highest mortality rates [3]. Non-IPD infections also exert a heavy burden in adults—pneumococcal pneumonia alone leads to ∼150 000 adult hospitalizations each year, with $1.3 billion in direct medical costs [2].

Because many pneumococcal infections require antibiotic treatment, antimicrobial resistance (AMR) poses a significant challenge to the treatment of pneumococcal disease. Drug-resistant *S. pneumoniae* has been classified as a serious threat by the US Centers for Disease Control and Prevention (CDC) and is estimated to account for ∼900 000 infections (30% of all pneumococcal infections) and 3600 deaths annually [2]. A recent worldwide study found that *S. pneumoniae* was the fourth leading pathogen in terms of deaths attributable to or associated with AMR [4]. Antibiotic-resistant disease leads to delayed disease resolution, resulting in more office visits, increased hospitalizations, and higher treatment costs [5].

AMR to commonly used respiratory antibiotics is an important concern for *S. pneumoniae* infections. In 2018–2019, 39.5% of pneumococcal bacteria collected from 328 US ambulatory centers and hospitals were reported as resistant to macrolides, with resistance rates of 29.6% in blood isolates and 47.3% in respiratory isolates [6]. Slightly lower nonsusceptible (intermediate or resistant) rates were observed in data from the CDC's Active Bacterial Core surveillance (ABCs), which reported 2019 *S. pneumoniae* nonsusceptibility rates of 29.3% for erythromycin, 18.2% for trimethoprim/sulfamethoxazole, and 12.5% for tetracycline among sterile site isolates [3]. Perhaps even more alarming is the high rate of multidrug resistance among *S. pneumoniae* isolates. A 2017 study of 105 hospitals in the SENTRY US database reported that 16% of *S. pneumoniae* isolates were multidrug resistant (defined as intermediate or resistant to ≥3 drug classes) [7].

One effective strategy for reducing both pneumococcal disease and AMR infections is pneumococcal vaccination [8–12]. There are 2 types of pneumococcal vaccine available for use in the United States, the 23-valent pneumococcal polysaccharide vaccine (PPSV23) and pneumococcal conjugate vaccines (PCVs), which currently include PCV13, PCV15 (also referred to as V114), and PCV20 [13, 14]. PPSV23 is highly effective in adults but is not indicated for children <2 years of age because their immune systems do not mount a robust response to polysaccharide antigens [15].

PCVs were subsequently developed to provide an effective immunization option for young children [13]. In 2010, the Food and Drug Administration (FDA) approved PCV13 for use in children and in 2012 expanded the indication to adults age >50 years [13], resulting in significant reductions in hospitalizations for pneumonia and lower respiratory tract infections in adults over 65 years of age [16]. The latest PCVs, which were approved by the FDA in 2021 for adults 18 years of age or older, provide broader serotype coverage (PCV15 [17] and PCV20 [18]). The Advisory Committee on Immunization Practices currently recommends the use of PCV20 alone or PCV15 in series with PPSV23 for all adults age ≥65 years and adults age 19–64 years with certain underlying conditions or risk factors [14].

A number of studies have shown that PCV use in the United States is associated with reduced AMR in *S. pneumoniae* [8–12]. Following introduction of PCV7, penicillin-nonsusceptible IPD rates declined by 81% in children under the age of 2 and by 49% in adults age ≥65 years [8], and the inclusion of additional serotypes in PCV13 further reduced AMR rates in both children and adults [10]. A recent international meta-analysis estimated that, overall, PCVs decreased the incidence of nonsusceptible pneumococcal infections by 56.9% [11]. Several factors may contribute to reduced AMR associated with vaccination, including inhibition of bacterial growth during established infection and reduction in antibiotic use due to overall decreases in respiratory infections [13, 19].

Despite the favorable effects of pneumococcal vaccines on reducing AMR, resistance to antibiotics persists in US *S. pneumoniae* isolates [2], in part due to increases in pneumococcal disease caused by serotypes not covered by PCVs, which are in some cases highly antibiotic resistant [8, 20]. Older adults are particularly affected: A recent study found that the incidence of IPD cases caused by antibiotic-resistant nonvaccine serotypes had more than tripled in adults age ≥65 years between 1998 and 2018 [20]. Other factors contributing to AMR include low pneumococcal vaccination rates, particularly in adults [21, 22], and high antibiotic usage rates for common respiratory antibiotics, including inappropriate prescriptions [23–25]. Several recent studies have documented increased AMR in North American *S. pneumoniae* isolates in both hospitalized and ambulatory adults [6, 7, 26–28].

These reports on high and increasing levels of resistance in *S. pneumoniae* isolates in the United States highlight the need for current information on AMR trends to help guide clinical management of pneumococcal infections and inform strategies designed to reduce resistance, including antimicrobial stewardship and vaccination campaigns. The objective of this study was to assess AMR trends in *S. pneumoniae* isolates collected from adults with pneumococcal disease.

## METHODS

### Study Design

This was a retrospective study of antibiotic susceptibility of nonduplicate *S. pneumoniae* isolates (first noncontaminant *S. pneumoniae* isolate within 30 days) collected from hospitalized and ambulatory adult patients (age ≥18 years) between January 2011 and February 2020. IPD was defined as cases with *S. pneumoniae* isolates obtained from cerebrospinal fluid (CSF)/neurology samples or blood (including valve and ventricle catheter tip sources). Non-IPD was defined as cases with *S. pneumoniae* isolates obtained from respiratory or ear/nose/throat (ENT) samples. Skin/wound, urine, and other nonsterile sources not listed above were evaluated but excluded from statistical modeling as they are not commonly associated with *S. pneumoniae* infections. Results likely to be associated with colonization (including environmental/surveillance specimens such as rectal or nasal swabs) or contamination were excluded by use of a previously described algorithm [29].

Reporting institutions consisted of US hospitals in the BD Insights Research Database (Becton, Dickinson and Company, Franklin Lakes, NJ, USA), which includes small and large hospitals in urban and rural areas, provides geographical representation across the United States, and contains electronically captured laboratory, pharmacy, patient demographics, administrative data, and admission, discharge, and transfer data feeds [2, 6, 26]. The study was approved as involving use of a limited retrospective data set for an epidemiology study and was exempt from consent by the New England Institutional Review Board/Human Subjects Research Committee (Wellesley, MA, USA) and conducted in compliance with Health Insurance Portability and Accountability Act requirements.

AMR was evaluated in *S. pneumoniae* isolates based on local facility laboratory reports from associated integrated delivery/health systems and standalone facilities; outpatient commercial laboratory companies were not included. Each laboratory applied local minimal inhibitory concentration (MIC) breakpoints for resistance; breakpoints were not standardized across facilities. Antibiotic resistance was assessed based on facility-reported interpretations using the following definitions:

- Penicillin resistant: intermediate (I) or resistant (R) to penicillin.
- Macrolide resistant: I or R to erythromycin, azithromycin, or clarithromycin.
- Fluoroquinolone resistant: I or R to levofloxacin or moxifloxacin.
- Extended-spectrum cephalosporin (ESC) resistant: I or R to ceftriaxone, cefotaxime, or cefepime.
- Tetracycline resistant: I or R to doxycycline or tetracycline.
- Any drug, 1, 2, or ≥3 drug resistant: I or R to any of the tested antibiotics, only 1, only 2, or ≥3 of the drugs listed above, respectively.

### Outcomes

For each category of resistance defined above, we evaluated the percentage of resistance (mean number of resistant isolates per total isolates tested) overall, by IPD vs non-IPD, year, sex, age, and hospital characteristics, including bed size, urban/rural location, teaching status, and geographic location based on US Census divisions [30].

### Statistical Analysis

Descriptive statistics of percentages of resistant isolates over time were presented by cross-tabulation. For multivariate analyses, the generalized estimating equations (GEE) method and logistic regression with a first-order autoregressive variance–covariance matrix were used to evaluate the quarterly trends of percent AMR. In the GEE framework, the time series data (count of resistant isolates) were viewed as repeated measures and hospital/facility modeled as random effect. These analyses were performed for resistance type as well as for 1, 2, and ≥3 drug resistance as mutually exclusive groups. All analyses were also stratified by invasive and noninvasive source type. Key additional factors included in the analyses were setting, age group, sex, and source type (blood, CSF/neurological, respiratory, and ENT). Multivariate analyses were controlled for hospital demographics, including bed size, urban/rural location, teaching status, and geographic region. All statistical analyses were conducted using R, version 4.0.3 (R Core Team 2020), and the R geepack package. *P* values <.05 were considered statistically significant.

## RESULTS

A total of 290 hospitals provided data from January 2011 to February 2020. The number of hospitals contributing data over the years ranged from 98 in 2011 to 259 in 2019 (Supplementary Table 1). Urban facilities accounted for 62.4% of facilities, and the West South Central, East South Central, and Middle Atlantic Census regions had the highest proportions of hospitals (17.9%, 17.2%, and 16.9%, respectively) (Supplementary Table 1).

### *S. pneumoniae* IPD and Non-IPD Isolates

A total of 34 039 nonduplicate *S. pneumoniae* isolates had susceptibility data over the study period and were included in analyses. Most (21 874; 64.3%) were derived from cultures collected at hospital admission; the remainder were collected in the ambulatory setting (8806; 25.9%) or during hospitalization (3359; 9.9%). Of the 34 039 *S. pneumoniae* isolates, 13 290 (39.0%) *S. pneumoniae* isolates were categorized as IPD and 20 749 (61.0%) as non-IPD (Table 1). Blood was the most common source for IPD (96.6% of IPD cases), and respiratory samples were the most common source for non-IPD (83.7% of non-IPD cases). The age group of 50–64 years had the most *S. pneumoniae* isolates for both IPD (34.6%) and non-IPD (34.8%) (Table 1).

**Table 1.** Demographics and Distribution of 30-Day Nonduplicate *S.*  *pneumoniae* Isolates With Susceptibility Data

Characteristic	IPD		Non-IPD		Total	
	No.	%	No.	%	No.	%
Overall	13 290	100	20 749	100	34 039	100
Age group						
18–34 y	878	6.6	1980	9.5	2858	8.4
35–49 y	1813	13.6	2941	14.2	4754	14.0
50–64 y	4604	34.6	7215	34.8	11 819	34.7
65–74 y	2778	20.9	4855	23.4	7633	22.4
>74 y	3217	24.2	3758	18.1	6975	20.5
Sex						
Female	6498	48.9	8885	42.8	15 383	45.2
Male	6792	51.1	11 864	57.2	18 656	54.8
Isolate source						
Blood	12 838	96.6	…	…	12 838	37.7
CSF	433	3.3	…	…	433	1.3
Neurological	19	0.1	…	…	19	0.1
Respiratory	…	…	17 368	83.7	17 368	51.0
ENT	…	…	1685	8.1	1685	5.0
Wound	…	…	1159	5.6	1159	3.4
Urine	…	…	315	1.5	315	0.9
Other	…	…	222	1.1	222	0.7

Totals may not equal 100% due to rounding.

Abbreviations: CSF, cerebrospinal fluid; ENT, ear, nose, throat; IPD, invasive pneumococcal disease.

### Antimicrobial Resistance in *S. pneumoniae* Isolates

**Figure 1.**
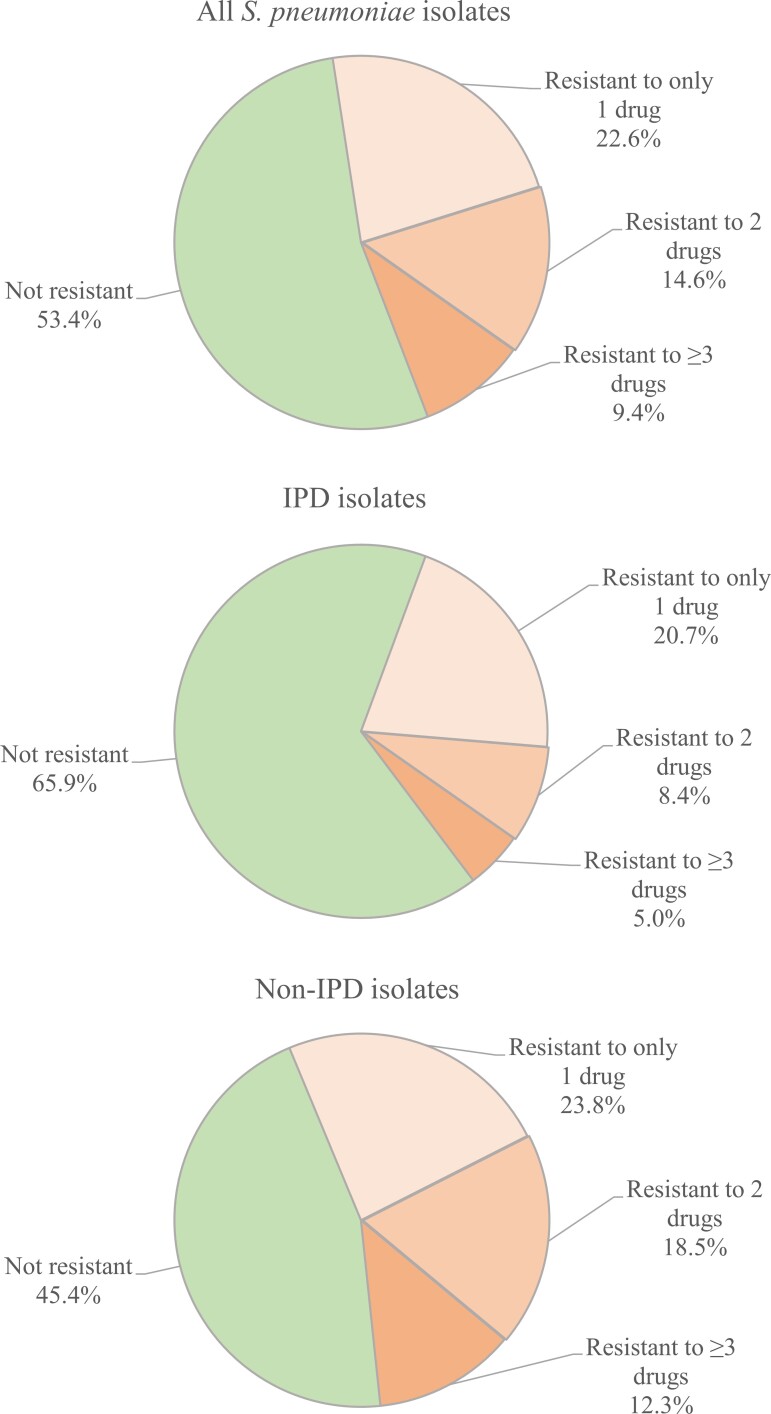
Resistance profiles of *S. pneumoniae* isolates by number of drugs based on observed data. Abbreviation: IPD, invasive pneumococcal disease.

During the study period, approximately half (15 850/34 039 [46.6%]) of *S. pneumoniae* isolates were resistant (I or R) to ≥1 drug. Resistance to 1 drug only (76 979/15 850 [48.4%]) was the most frequent phenotype among resistant isolates, but resistance to 2 drugs (4961/15 850 [31.3%]) and ≥3 drugs (3210/15 850 [20.3%]) was also common (Table 2, Figure 1). The highest rate of resistance was observed for macrolides (37.7%), followed by penicillin (22.1%) and tetracyclines (16.1%) (Table 2).

**Table 2.** Antimicrobial Resistance in *S. pneumoniae* Isolates in Adults (January 2011 to February 2020)

Antibiotic	IPD (n = 13 290)	Non-IPD (n = 20 749)	Total (n = 34 039)
Resistance to ≥1 drug	4528 (34.1)	11 322 (54.6)	15 850 (46.6)
Resistance to only 1 drug	2748 (20.7)	4931 (23.8)	7679 (22.6)
Resistance to 2 drugs	1114 (8.4)	3847 (18.5)	4961 (14.6)
Resistance to ≥3 drugs	666 (5.0)	2544 (12.3)	3210 (9.4)
Resistance by antibiotic class			
Macrolide	3609 (27.2)	9238 (44.5)	12 847(37.7)
Penicillin	1909 (14.4)	5607 (27.0)	7516 (22.1)
Tetracycline	1121 (8.4)	4346 (20.9)	5467 (16.1)
ESC	355 (2.7)	1144 (5.5)	1499 (4.4)
Fluoroquinolone	105 (0.8)	493 (2.4)	598 (1.8)

Data are presented as No. (%). Totals may not equal 100% due to rounding.

Abbreviations: ESC, extended-spectrum cephalosporin; IPD, invasive pneumococcal disease.

Rates of resistance to ≥1 drug were higher for non-IPD (54.6%) compared with IPD isolates (34.1%) (Table 2). This pattern was also observed for all evaluated drug classes. Observed resistance rates were >2-fold higher for tetracycline (20.9% for non-IPD vs 8.4% for IPD) and nearly 2-fold higher for penicillin (27.0% vs 14.4%). Non-IPD isolates also had higher rates of resistance to 2 drugs (18.5% vs 8.4%) and ≥3 drugs (12.3% vs 5.0%) (Table 2).

*S. pneumoniae* blood isolates had significantly higher rates of resistance to macrolides, fluoroquinolones, and tetracyclines compared with CSF/neurological isolates, and respiratory isolates had significantly higher rates of resistance to macrolides, fluoroquinolones, ESC, and tetracyclines compared with ENT isolates.

### Influence of Demographic and Hospital Characteristics on AMR

In multivariate-adjusted analyses, older age, non-IPD isolate source, and geographic location were consistently associated with significant differences in AMR rates (Table 3; Supplementary Table 2). Geographic locations with the highest resistance varied by antibiotic class. The West North Central had the highest rates of macrolide and penicillin resistance, whereas the South Atlantic had the highest rates of tetracycline, fluoroquinolone, and ESC resistance (Supplementary Table 2). Calendar quarter was associated with significant differences for antibiotic classes, with the exception of tetracycline (Supplementary Table 2), indicating seasonal fluctuations in *S. pneumoniae* AMR as previously reported [26]. Hospital onset of tested cultures (vs ambulatory or admission) was also associated with significant differences for most antibiotics. Other characteristics, such as sex and hospital size, were not consistently associated with significant differences in resistance rates (Supplementary Table 2).

**Table 3.** Adjusted^a^  *S. pneumoniae* Resistance Rates (95% CI) by Demographic and Hospital Characteristics

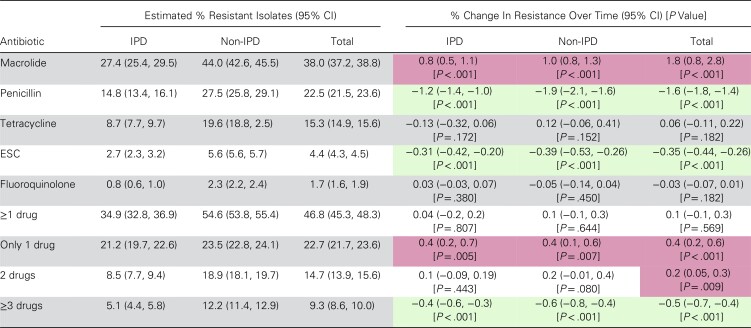

Abbreviations: CSF, cerebrospinal fluid; ENT, ear, nose, throat; IPD, invasive pneumococcal disease.

a Models were adjusted by age, gender, setting, quarter, hospital demographics, and US Census region. Additional drugs and variables are presented in Supplementary Table 2.

### Trends in AMR Over Time

Adjusted rates of *S. pneumoniae* resistance to the 3 antibiotics with the highest number of resistant isolates showed varying patterns over time (Table 3). From January 2011 to February 2020, macrolide resistance increased for IPD (20.7%–28.2%; *P* < .001), non-IPD (38.3%–46.7%; *P* < .001), and all (IPD plus non-IPD) isolates (31.7%–40.2%; *P* < .001). In contrast, penicillin resistance rates decreased over time, and tetracycline resistance rates did not show significant fluctuations. ESC and fluoroquinolone resistance rates decreased over time (Supplementary Table 2).

Multivariate modeling confirmed a significant increasing overall trend in macrolide resistance for IPD (+0.8% per year), non-IPD (+1.0% per year), and total *S. pneumoniae* isolates (+1.8% per year; all *P* values < .001) (Table 4). Significant decreasing trends were observed in total *S. pneumoniae* isolates for penicillin (−1.6% per year; *P* < .001) and ESC (−0.35% per year; *P* < .001) and occurred in both IPD and non-IPD isolates. Resistance to fluoroquinolones and tetracyclines did not show significant fluctuations during the study time period (Table 4). Resistance to ≥1 drug stayed constant during the study. Increasing trends were observed for resistance to only 1 drug (+0.4% per year; *P* < .001) and to 2 drugs (+0.2% per year; *P* = .009) for total isolates, while resistance to ≥3 drugs significantly decreased (−0.5% per year; *P* < .001). For IPD and non-IPD isolates, increasing trends in resistance to ≥1 drug and only 1 drug and the decreasing trend in resistance to ≥3 drugs were retained, but changes in 2-drug resistance did not achieve significance.

**Table 4.** Model-Estimated^a^ Rates of Resistance and Annual Percent Change in *S. pneumoniae* AMR in Isolates Collected From US Adults (January 2011 to February 2020)

Antibiotic	Estimated % Resistant Isolates (95% CI)			% Change In Resistance Over Time (95% CI) [*P* Value]		
	IPD	Non-IPD	Total	IPD	Non-IPD	Total
Macrolide	27.4 (25.4, 29.5)	44.0 (42.6, 45.5)	38.0 (37.2, 38.8)	0.8 (0.5, 1.1)[*P* < .001]	1.0 (0.8, 1.3)[*P* < .001]	1.8 (0.8, 2.8)[*P* < .001]
Penicillin	14.8 (13.4, 16.1)	27.5 (25.8, 29.1)	22.5 (21.5, 23.6)	−1.2 (−1.4, −1.0)[*P* < .001]	−1.9 (−2.1, −1.6) [*P* < .001]	−1.6 (−1.8, −1.4) [*P* < .001]
Tetracycline	8.7 (7.7, 9.7)	19.6 (18.8, 2.5)	15.3 (14.9, 15.6)	−0.13 (−0.32, 0.06) [*P* = .172]	0.12 (−0.06, 0.41) [*P* = .152]	0.06 (−0.11, 0.22) [*P* = .182]
ESC	2.7 (2.3, 3.2)	5.6 (5.6, 5.7)	4.4 (4.3, 4.5)	−0.31 (−0.42, −0.20) [*P* < .001]	−0.39 (−0.53, −0.26) [*P* < .001]	−0.35 (−0.44, −0.26) [*P* < .001]
Fluoroquinolone	0.8 (0.6, 1.0)	2.3 (2.2, 2.4)	1.7 (1.6, 1.9)	0.03 (−0.03, 0.07) [*P* = .380]	−0.05 (−0.14, 0.04) [*P* = .450]	−0.03 (−0.07, 0.01) [*P* = .182]
≥1 drug	34.9 (32.8, 36.9)	54.6 (53.8, 55.4)	46.8 (45.3, 48.3)	0.04 (−0.2, 0.2) [*P* = .807]	0.1 (−0.1, 0.3) [*P* = .644]	0.1 (−0.1, 0.3) [*P* = .569]
Only 1 drug	21.2 (19.7, 22.6)	23.5 (22.8, 24.1)	22.7 (21.7, 23.6)	0.4 (0.2, 0.7) [*P* = .005]	0.4 (0.1, 0.6) [*P* = .007]	0.4 (0.2, 0.6) [*P* < .001]
2 drugs	8.5 (7.7, 9.4)	18.9 (18.1, 19.7)	14.7 (13.9, 15.6)	0.1 (−0.09, 0.19) [*P* = .443]	0.2 (−0.01, 0.4) [*P* = .080]	0.2 (0.05, 0.3) [*P* = .009]
≥3 drugs	5.1 (4.4, 5.8)	12.2 (11.4, 12.9)	9.3 (8.6, 10.0)	−0.4 (−0.6, −0.3) [*P* < .001]	−0.6 (−0.8, −0.4) [*P* < .001]	−0.5 (−0.7, −0.4) [*P* < .001]

Red shading indicates a significant increase in resistance rate over time; green shading indicates a significant decrease.

Abbreviations: AMR, antimicrobial resistance; ESC, extended-spectrum cephalosporins; IPD, invasive pneumococcal disease.

a Models were adjusted by age, gender, setting, quarter, hospital demographics, and US Census region.

## DISCUSSION

Our analysis of >34 000 *S. pneumoniae* isolates from adults throughout the United States between January 2011 and February 2020 indicates that *S. pneumoniae* AMR rates remain high. Although slight decreasing trends in resistance to beta-lactam agents (penicillin and ESC) and ≥3 drugs are encouraging, increasing macrolide resistance rates and the consistent high rates of resistance to ≥1 drug indicate the need for continued attention to efforts to reduce and treat antibiotic-resistant *S. pneumoniae* infections.

The finding of high levels of macrolide resistance is consistent with previous reports on *S. pneumoniae* resistance in hospitalized [3, 7, 26, 27] and ambulatory [3, 26] US adults and with a report on increasing macrolide resistance in *S. pneumoniae* in Canadian hospitals from 2007 to 2016 [28]. International surveillance studies indicate that elevated levels of *S. pneumoniae* macrolide resistance are also a significant concern outside of North America, [31, 32]. Macrolides are frequently used to treat respiratory conditions, and current American Thoracic Society/Infectious Disease Society of America guidelines recommend macrolides as first-line therapy for outpatients with CAP if local pneumococcal resistance is <25% [33]. However, this recommendation has been called into question [34] based on the high levels of macrolide resistance in both inpatient and outpatient settings throughout the United States [6]. In outpatient CAP, macrolide failure is associated with poor outcomes and increased hospital costs [35].

The stable pattern of resistance to ≥1 drug masked the off-setting trends of increased resistance to only 1 drug and to 2 drugs vs decreasing resistance to ≥3 drugs. These data indicate that *S. pneumoniae* isolates in the United States are becoming less highly resistant, which could translate into a greater likelihood of effective options in patients failing initial therapy. A study based on data from 28 US medical centers in the Assessing Worldwide Antimicrobial Resistance Evaluation (AWARE) Program identified similar encouraging trends in reduced resistance to ≥3 drugs between 2011 and 2020 [27].

We note with interest the higher rates of *S. pneumoniae* drug resistance and multidrug resistance in non-IPD vs IPD isolates, an observation previously made in a study conducted in Korea [36]. Because of its generally lower clinical severity, non-IPD has not been as thoroughly studied as IPD, and in the United States these cases are not tracked by the CDC [37]. Nevertheless, the burden of non-IPD, particularly CAP, is substantial. A recent international meta-analysis estimated that in the period 1–5 years after introduction of PCV10/PCV13 vaccines, ∼18% of CAP continued to be due to pneumococcal disease; about half of the cases (49%) were associated with PCV13 serotypes [38]. In the United States, an estimated 569 260 cases of nonbacteremic CAP occur annually in adults ≥50 years of age, resulting in estimated medical and indirect costs of >$4 billion in 2013 US dollars [39]. Of potentially even greater importance, non-IPD cases account for a significant amount of antibiotic use [40], thereby potentially contributing to antibiotic resistance.

As with overall IPD cases in the United States [37], AMR in adult pneumococcal disease is driven largely by serotypes not covered by PCV13 [7, 10, 41, 42], and this trend is most pronounced in adults ≥65 years of age [20]. The introduction of PCV vaccines has been associated with significant reductions in pneumococcal disease and in *S. pneumoniae* AMR [8–12], and hypothetical models support additional benefits with new PCVs recently approved for use in adults [43, 44]. However, current adult pneumococcal vaccination rates in the United States are low. Based on 2018 National Health Interview Survey data, pneumococcal vaccination rates are 23.3% in US adults at increased risk of pneumococcal disease and 69.0% in adults 65 years of age or older [21]. Both rates are far below the CDC 2020 Healthy People goals of 60% and 90%, respectively [45]. Although pneumococcal vaccination is currently not recommended for low-risk adults age 50–64 [14], our results show that this age group had the most *S. pneumoniae* isolates for both IPD (34.6%) and non-IPD (34.8%), suggesting that this population may also benefit from pneumococcal vaccination. The US National Vaccine Advisory Committee [46] and the World Health Organization [47] advocate the increased use of vaccination as a means of reducing AMR worldwide.

Even with improved vaccination rates, however, pneumococcal disease caused by resistant *S. pneumoniae* is unlikely to disappear. Despite overall reduction in cases caused by AMR *S. pneumoniae*, wider adoption of pneumococcal vaccination, particularly in children, has been associated with the emergence of drug-resistant serotypes not covered by the vaccine [48, 49], leading some experts to liken attempts to eradicate key pneumococcal serotypes to a “whack a mole” game [50]. Other important components in combatting AMR include antimicrobial stewardship efforts and the development of new drugs that effective against antibiotic-resistant *S. pneumoniae* [4, 51].

The limitations of our study include analyses of resistance based on local microbiology practices at each facility, which may have influenced reported resistance rates. In particular, the penicillin resistance rates observed in our study are higher than those reported in a recent US study (4.0%) [7] or by the CDC ABCs (3.6%) [3], both of which used central laboratories for testing. This discrepancy may relate to differences in the patient populations/geographic locations or to a change in penicillin breakpoints introduced by the Clinical and Laboratory Standards Institute (CLSI) in 2013, in which the nonmeningitis nonsusceptibility breakpoint for parenteral penicillin was increased from an MIC of >0.06 mg/L to >2 mg/L [52]. In a recent analysis of US *S. pneumoniae* isolates (n = 7901), penicillin nonsusceptible rates were 38.8% using the meningitis/oral penicillin breakpoint (>0.06 mg/L) and 6.2% using the parenteral penicillin breakpoint (>2 mg/L) [27]. CLSI changes can take many years to be fully adopted at the clinical laboratory level [53], so it is possible that delayed adoption of this CLSI change inflated the percentage of penicillin-resistant *S. pneumoniae* isolates reported by facilities in our database. It is important to note, however, that the data presented here reflect information available to clinicians during daily management of their patients. Other potential limitations of our study include lack of information on associated infections; our analyses were based solely on culture-positive isolates, and the presence of symptomatic pneumococcal disease was not confirmed. We did not evaluate the primary source of bacteremia. As with all microbiologic surveillance studies, selection bias is a potential issue due to a higher likelihood of testing more severely ill patients, which may increase estimates of resistance. Our study was not designed to evaluate the pneumococcal vaccination status of patients or *S. pneumoniae* serotypes. These data would be valuable additions to a future analysis.

In conclusion, our findings document continued high rates of AMR in *S. pneumoniae* isolates from adults with IPD or non-IPD as well as increasing trends in resistance for macrolides. These data may be valuable in informing both treatment decisions and campaigns to reduce AMR, including increased pneumococcal vaccination and targeted antimicrobial stewardship programs.

## Supplementary Material

ofac420_Supplementary_Data

## Supplementary Data

Supplementary materials are available at *Open Forum Infectious Diseases* online. Consisting of data provided by the authors to benefit the reader, the posted materials are not copyedited and are the sole responsibility of the authors, so questions or comments should be addressed to the corresponding author.
