# Supplementary material for: A Multicenter Evaluation of Trends in Antimicrobial Resistance Among Streptococcus pneumoniae Isolates From Adults in the United States
Source: Open Forum Infect Dis. 2022 Sep 2;9(9):ofac420. doi: 10.1093/ofid/ofac420 (PMC9511122; doi:10.1093/ofid/ofac420)
Supplement: ofac420_Supplementary_Data [file ofac420_supplementary_data.zip › 2022 06 15 Adult pneumonia Suppl Table 1.docx]

**Supplementary Materials**

**Supplemental Table 1. Hospital Demographics**

| **Characteristic** | **Number of hospitals** | |
| --- | --- | --- |
|  | **n** | **%** |
| **Total** | 290 | 100.0 |
| **Year**^a^ |  |  |
| 2011 | 98 | 33.8 |
| 2012 | 106 | 36.6 |
| 2013 | 117 | 40.3 |
| 2014 | 151 | 52.1 |
| 2015 | 172 | 59.3 |
| 2016 | 191 | 65.9 |
| 2017 | 212 | 73.1 |
| 2018 | 248 | 85.5 |
| 2019 | 259 | 89.3 |
| 2020 (Q1) | 254 | 87.6 |
| **Bed size** |  |  |
| Less than 100 | 102 | 35.2 |
| 100-300 | 116 | 40.0 |
| Greater than 300 | 72 | 24.8 |
| **Urban/Rural** |  |  |
| Rural | 109 | 37.6 |
| Urban | 181 | 62.4 |
| **Teaching status** |  |  |
| Non-teaching | 189 | 65.2 |
| Teaching | 101 | 34.8 |
| **Census region**^b^ |  |  |
| East North Central | 42 | 14.5 |
| East South Central | 50 | 17.2 |
| Middle Atlantic | 49 | 16.9 |
| Mountain | 6 | 2.1 |
| New England | 5 | 1.7 |
| Pacific | 33 | 11.4 |
| South Atlantic | 43 | 14.8 |
| West North Central | 10 | 3.4 |
| West South Central | 52 | 17.9 |

Totals may not equal 100% due to rounding.

^a^ The number of hospitals varied by year; the total of 290 reflects all facilities that contributed data in any year.

^b^ States included in the data sample in the designated census regions were:

East North Central: Illinois, Indiana, Michigan, Ohio, and Wisconsin

East South Central: Alabama, Kentucky, Mississippi, and Tennessee

Middle Atlantic: New Jersey, New York, and Pennsylvania

Mountain: Arizona, Idaho, Montana, and New Mexico

New England: Connecticut and New Hampshire

Pacific: California, Oregon, and Washington

South Atlantic: Delaware, Georgia, Florida, Maryland, North Carolina, South Carolina, Washington D.C., West Virginia, and Virginia

West North Central: Iowa and Missouri

West South Central: Louisiana, Oklahoma, Texas
